# Breast Cancer: A Multi-Disciplinary Approach from Imaging to Therapy

**DOI:** 10.3390/curroncol31010043

**Published:** 2024-01-22

**Authors:** Daniele Ugo Tari

**Affiliations:** Department of Breast Imaging, Caserta Local Health Authority, District 12 “Palazzo della Salute”, 81100 Caserta, Italy; daniele.tari@aslcaserta.it

## 1. Introduction

Breast cancer (BC) is the most prevalent form of cancer among women worldwide, accounting for over 2 million diagnoses annually [1]. The impact of BC extends beyond individual patients, affecting the entire community through its implications in imaging, therapy, and its broader social, economic, and psychological consequences. In the face of ongoing challenges, a comprehensive approach is crucial to ensuring a high standard of care, particularly in the aftermath of the COVID-19 pandemic, which has brought lasting changes to medical practices [2]. The demand for personalized medicine has underscored the importance of a multidisciplinary strategy combining artificial intelligence and human expertise [3].

Early detection of breast cancer has significantly improved survival rates, enabling more effective and targeted treatments. However, a substantial gap persists when early diagnosis is not achieved, particularly among women with dense breasts or those at high risk [4,5]. Additionally, the incidence of male breast cancer has risen by 20–25% in recent decades [6]. Consequently, a multidisciplinary team and enhanced diagnostic-therapeutic pathways (DTCP) are essential for early detection and improved treatments [7].

The primary aim of this Special Issue has been to comprehensively present and discuss all aspects of breast cancer management, from imaging to therapy, addressing knowledge gaps, and exploring the psychological aspects of diagnosis and therapy. Out of nineteen submitted articles, eleven were accepted for publication after the peer-review process, resulting in a 58% acceptance rate. The published articles, briefly described in the following section, cover several topics and aspects significantly influencing breast cancer management.

## 2. Summary of Published Articles

In the first article of this Special Issue, Bhardwaj et al. (Contribution 1) assessed the efficacy and coordination of a multidisciplinary team (MDT) in the treatment of early-stage breast cancer using neoadjuvant chemotherapy (NAC). The retrospective study covered 94 patients and focused on the timing and outcomes of NAC, surgery, and radiation therapy. The study found significant downstaging of breast tumors in 91.4% of patients and axillary downstaging in 33% of patients. The median time from diagnosis to NAC was 37.5 days, from the end of NAC to surgery was 29 days, and from surgery to radiation therapy was 49.5 days. The study concluded that the MDT provided timely, coordinated, and consistent care, with the time to treatment aligning with national trends. It highlighted the effectiveness of multidisciplinary coordination in managing early-stage breast cancer, suggesting this as a model for other community cancer centers.

Muradali et al. (Contribution 2) synthesized the inconsistencies in the use of preoperative breast magnetic resonance imaging (MRI) following the diagnosis of breast cancer through mammography and/or ultrasound. After conducting a systematic review and meta-analysis, they recommended considering preoperative breast MRI on a case-by-case basis, especially for patients where additional information about disease extent could influence treatment decisions. The study substantiated that MRI improves recurrence rates, decreases reoperations, and increases detection of synchronous contralateral breast cancer. It emphasized the need for shared decision-making between care providers and patients, considering the benefits and risks of MRI as well as patient preferences. Specific recommendations are given for using MRI in various clinical scenarios, such as aiding surgical planning, identifying lesions in dense breasts, and determining the presence of muscle or chest wall invasion in certain tumors. The paper underlined the significance of MRI’s high sensitivity and specificity in breast cancer diagnosis and staging, advocating for its selective use in enhancing treatment outcomes.

The study proposed by D’Angelo et al. (Contribution 3) is a retrospective investigation into the use of Magseed^®^ (Endomagnetics, Cambridge, UK) for the preoperative localization of non-palpable breast lesions. It involved 45 patients who underwent breast-conserving surgery (BCS) between 2020 and 2022, with Magseed placement primarily under ultrasound guidance. The study boasted a high placement success rate of 97.8%, with only one instance of seed migration, and a 100% retrieval rate post-BCS. Notably, no patients required re-excision due to positive margins. The authors concluded that Magseed is an extremely effective technique for preoperative localization of non-palpable breast lesions, supporting its continued use despite acknowledging certain limitations and rare occurrences of seed migration. The study contributed valuable real-world data to the growing body of literature on Magseed, suggesting it as a viable alternative to traditional wire localization techniques.

Malainou et al. (Contribution 4) delved into the unique challenges and clinical implications of estrogen receptor-low-positive (ER-low-positive) breast cancer. This subtype, characterized by 1–9% ER expression, represents a small but significant portion of breast cancer cases, and its management is less clear-cut due to its nuanced response to standard therapies. The review consolidated various studies that discuss the prevalence, characteristics, and treatment responses of ER-low-positive BC. It highlighted that while these tumors share some similarities with ER-negative and triple-negative breast cancers, they present distinct clinical behaviors and outcomes. The review called for more research, especially randomized clinical trials, to better understand and manage this unique breast cancer subset, emphasizing the need for tailored treatment strategies and the potential of including these patients in clinical trials for more aggressive breast cancer types. This review is a call to action for the medical community to recognize the distinct nature of ER-low-positive BC and to seek more effective management strategies.

Oliveira et al. (Contribution 5) presented an observational analysis of 69 BRCA1/2 ovarian cancer survivors and their subsequent risk of developing breast cancer. The authors found that the median overall survival after ovarian cancer diagnosis was 8 years, with a significantly higher survival rate for BRCA2 patients compared to BRCA1 patients. About 13.2% of the participants developed breast cancer at a median age of 61 years. The study discussed the controversy of risk-reducing bilateral breast surgery in ovarian cancer survivors due to the associated high relapse rates and mortality of ovarian cancer. While the study acknowledged the potential benefits of surgical breast cancer risk management, it emphasized that such decisions should be tailored to individual patient characteristics and preferences, considering the balance between ovarian cancer mortality and breast cancer risk. This study contributes to the ongoing discourse on the management of cancer risks in BRCA1/2 ovarian cancer survivors, suggesting a multidisciplinary approach to decision-making.

Casella et al. (Contribution 6) presented a study exploring the clinical and aesthetic outcomes of immediate versus delayed symmetrization in skin-reducing mastectomy (SRM) for BC. The study involved a randomized observational cohort of 84 patients undergoing SRM, divided into two groups: immediate and delayed symmetrization. The study found that immediate symmetrization provided better aesthetic outcomes and higher patient satisfaction without significantly impacting the second stage of reconstruction. It highlighted immediate symmetrization as a safe and tolerable technique, improving the quality of life for patients. The research underscored the importance of considering immediate symmetrization in reconstructive surgery planning for breast cancer patients to provide better immediate symmetry and overall satisfaction.

The next two papers evaluated the impact of radiotherapy (RT) on right and left BC, respectively. In particular, Guzeloz et al. (Contribution 7) explored the relationship between radiotherapy dose-volume parameters for right breast cancer and subsequent changes in liver function tests (LFTs), specifically alanine aminotransferase (ALT), aspartate aminotransferase (AST), and gamma-glutamyl transferase (GGT). The retrospective analysis included 100 female patients treated across three centers, focusing on liver dosimetry during right breast or chest wall RT and its impact on LFTs pre- and post-RT. The results showed a median increase of up to 15% in AST, ALT, and GGT levels post-radiotherapy, with a significant correlation between higher liver doses and changes in LFTs. The study emphasized the importance of considering liver dose during radiotherapy planning and the necessity of regular LFT monitoring, advocating for a mean liver dose below 208 cGy to minimize potential liver damage. The study contributed to understanding the implications of RT on liver function and underscored the need for careful dose management to prevent liver toxicity, particularly in breast cancer patients with a longer life expectancy.

Antunac K et al. (Contribution 8) investigated the relationship between radiation doses to cardiac structures and the elevation of high-sensitivity cardiac troponin I (hscTnI) as an early marker of cardiotoxicity in patients receiving adjuvant radiotherapy for left-sided breast cancer along with anti-HER2 therapy. Including 61 patients, the study found that patients with an increase in hscTnI values post-radiotherapy had significantly higher mean radiation doses to the heart, left ventricle (LV), and left anterior descending artery (LAD) compared to those without an hscTnI increase. The findings suggested that higher radiation doses to these cardiac structures are associated with subclinical myocardial damage, as indicated by elevated hscTnI levels. The study underscored the importance of optimizing radiation therapy techniques to minimize cardiac exposure and the potential for early cardiac injury in breast cancer treatment.

The last three articles explored the psychological impact that a diagnosis or a potential diagnosis of breast cancer can have on women, assessing its effects on both an individual and familial level.

Oprean C et al. (Contribution 9) proposed a poignant case study of a 31-year-old woman grappling with metastatic HER2-positive breast cancer who chose to pursue pregnancy despite the known risks. The patient initially responded well to first-line treatment but vehemently refused ongoing oncological care due to her strong desire to conceive. This decision led to the cessation of treatment and the subsequent progression of her disease. Unfortunately, her pregnancy and life ended abruptly due to complications from her cancer. This case underscored the complex psychological aspects influencing patient decisions, including cognitive distortion, which led to prioritizing procreation over personal survival. The study emphasized the importance of multidisciplinary care and psychological support in managing such challenging cases, highlighting the urgent need for careful guidance and support for patients making life-altering decisions under the weight of severe illness.

Isselhard et al. (Contribution 10) provided a comprehensive evaluation of the psychological distress experienced by women who carry BRCA1/2 pathogenic variants but are not affected by cancer. This systematic review included 45 studies from 13 countries focusing on measures of distress, depression, and other psychological outcomes. Most studies observed an initial peak in distress following the disclosure of genetic test results, which tended to decline over subsequent months. While depression was frequently investigated, it was generally not found to be clinically significant among carriers. Quality of life appeared largely unaffected, though younger women showed some dissatisfaction with their role functioning. Body image was less frequently assessed, but available evidence suggested a decrease in body image satisfaction, especially after prophylactic mastectomy. The review called for future research to use standardized instruments to enhance comparability and provide more definitive conclusions about the psychological morbidity in this specific population.

Finally, Leite et al. (Contribution 11) delved into the experiences of violence endured by women from their intimate partners following mastectomy. Conducted with 16 Brazilian women who underwent breast cancer treatment, this qualitative study revealed alarming insights into the types of violence—psychological, physical, and sexual—that these women faced during their already vulnerable post-mastectomy period. The results highlighted that 50% of participants encountered psychological violence, 30% physical violence, and 20% sexual violence from their intimate partners. The research underscored the pressing need for healthcare professionals to be vigilant and proactive in identifying and addressing intimate partner violence among mastectomized women, recognizing it as a significant factor affecting their overall treatment and recovery. The study’s conclusions advocated for a comprehensive, multidisciplinary approach to supporting these women, including the establishment of a protective and care network to combat the pervasive issue of violence.

## 3. Conclusions

The interdisciplinary nature of these discussions underscores the need for a holistic understanding and approach to breast cancer, emphasizing the importance of collaborative efforts in advancing knowledge and improving patient outcomes.

In particular, it would be desirable for scientific research to systematically compare practical experiences with extant literature, with the objective of providing empirically grounded guidance for clinical practice and meticulously embodying the principles of evidence-based medicine. Such a rigorous approach holds the potential to substantially contribute to the establishment of uniformity across diverse socio-economic contexts.

In conclusion, the imperative for further in-depth research and development remains crucial. It is vital to thoroughly understand and address the management of breast cancer diagnosis and treatment, facing new challenges with steadfast commitment and dedication.
